# Event detection via graph convolutional networks for multi-source multimodal integration

**DOI:** 10.1371/journal.pone.0344266

**Published:** 2026-07-29

**Authors:** Quanlong Fan, Gang Xu, Yunge Wang, Mengke Wu

**Affiliations:** 1 Zhejiang Cheng ‘an Big Data Co., LTD, Wenzhou, China; 2 Zhejiang College of Security Technology, College of New Energy Equipmen, Wenzhou, China; 3 Faculty of Artificial Intelligence, Zhejiang Institute of Security Vocational Technology, Wenzhou, China; Hiroshima University: Hiroshima Daigaku, JAPAN

## Abstract

In real-time event detection, social media platforms like Twitter, Instagram, and Weibo provide valuable data, where users share updates, opinions, and multimedia content. However, existing event detection algorithms typically rely on a single data source or modality, limiting their ability to process the complex, multimodal nature of event information. To address this, we propose a novel framework that integrates multi-source, multimodal data to improve event detection robustness. Using graph convolutional networks, the framework captures both textual and visual information, incorporating syntactic and dependency details. It employs cross-modal attention to combine features from text and images, enhancing the system’s understanding of how these modalities complement each other. The model identifies event trigger words in the text and classifies events based on both textual and visual cues. Our approach improves event detection accuracy and reliability. Evaluation using news data from the same period as social media posts shows that combining text, images, and news data increases event detection and classification accuracy, offering a more dynamic and comprehensive solution.

## Introduction

With the rapid development of information technology and the widespread adoption of internet-based social media, real-time processing of massive multi-source heterogeneous data has become a critical challenge. Platforms such as Weibo, WeChat, and Twitter generate petabytes of user-generated content daily, encompassing multiple modalities including text, images, with over 80% being unstructured data [1,2]. Traditional structured data processing methods struggle to handle this complexity, particularly in time-sensitive scenarios like event detection. In response, event extraction technology incorporating multimodal features has emerged, which employs deep learning models to automatically identify key event elements such as time, location, and participants from text and converts them into structured representations [3–5]. Research demonstrates that cross-modal approaches integrating visual features can significantly improve event recognition accuracy (by 15–20% compared to unimodal methods) while maintaining processing efficiency [6–9], providing reliable technical support for applications such as public opinion monitoring and emergency management.

The goal of event extraction is to transform event information from text into structured data, usually by extracting key elements of the event, such as “who, when, where, what, why, and how..” [10] By extracting these key pieces of information, event extraction helps us gain a comprehensive understanding of the background, process, and impact of events. For example, in news reports, event extraction systems can automatically identify information such as the time, location, participants, event type, and cause of the event, and convert this data into structured formats like tables or databases [11]. This structured data can facilitate subsequent data analysis and processing, and can be used in statistical analysis, trend prediction, and other applications. In addition, event extraction systems can help us discover potential important events within text, especially when dealing with vast amounts of social media data. For example, during the occurrence of a public emergency, a large number of posts, comments, and articles on social media can provide real-time updates and public sentiment feedback on the event. Through event extraction technology, key information related to the event can be automatically extracted, providing strong data support for emergency management, public opinion monitoring, and other areas.

Recent years have witnessed significant progress in single-source approaches for sentiment analysis, text classification, and event prediction, where these methods typically extract and predict information from a single data source (e.g., social media text) while enhancing performance through techniques like sentiment word mining and machine learning models. However, existing event detection technologies primarily focus on individual data sources (social media streams or news feeds) without adequately accounting for the distinct characteristics of different dissemination channels. In reality, substantial variations exist in event propagation speeds and acquisition channels – for instance, sudden incidents like the Tokyo earthquake spread rapidly through social media, while political events such as the Paris Climate Agreement are predominantly covered by traditional media outlets [12–14]. Although single-source methods have improved model performance by incorporating multimodal data or developing real-time processing frameworks [15], achieving accurate event detection still requires the development of novel models capable of adapting to the unique characteristics of different information sources.

To ensure the reliability and real-time capability of the event detection model in a multi-source data environment, we need to collect multilingual text streams from different channels as the input for the model. These channels include not only social media platforms (such as Twitter, Facebook, Weibo, etc.) and news websites (such as CNN, BBC, Xinhua News Agency, etc.), but also blogs, forums platforms, and other diverse sources of information. Each data source has distinct content characteristics, publication frequency, and dissemination patterns [16–19]. Therefore, establishing effective connections between these sources and ensuring that the model can handle both long and short texts from different origins is a new challenge [20,21].

In event detection tasks, social media texts are typically short, frequent, and highly unstructured, while news reports tend to be more formal, structured, and longer. Integrating multilingual information from different text sources and handling the varying text lengths become major challenges in building an efficient event detection model. To address these challenges, we propose a comprehensive event detection model framework that incorporates dependency information using Graph Convolutional Networks (GCNs). The framework consists of four core modules: the encoding layer, the graph construction module, the GCN layer that integrates dependency information, and the trigger word recognition and classification layer. Initially, word embeddings are pre-trained using the skip-gram model, converting each word in the input sentence into a fixed-length real-valued vector. Visual features are also extracted using a pre-trained CLIP model. The graph construction module builds multi-order syntactic graphs and dependency syntactic graphs based on the syntactic dependency tree of the sentence, where tokens serve as nodes and syntactic arcs as edges. The encoded sentence representations from the encoding layer and the outputs from the graph construction module are passed into the GCN for modeling. After passing through the GCN, the resulting output integrates both dependency label information and multi-hop relational data. Finally, in the trigger word recognition and classification layer, the final sentence representation vector, enhanced by dependency information from the GCN layer, is fed into a feed-forward neural network. This network, combined with a softmax function, classifies each word in the sentence, identifying the trigger words and categorizing them into respective event types. Compared to other recent event detection models based on Graph Convolutional Networks, the proposed method stands out by leveraging multi-order syntactic graphs and dependency graphs to handle multi-language, long and short texts, and cross-modal information more effectively. Moreover, the fusion of multimodal features (text and image) and the integration of GCNs significantly improve the model’s accuracy, especially when dealing with complex social media and news text, enabling the effective fusion of features from diverse sources of information.

The three main contributions of this method are as follows:

1. **Multimodal Feature Fusion**: By integrating multi-language information from different text sources through graph convolutional networks and handling the length differences in these text streams, this method achieves the fusion processing of different text sources such as social media text and news reports.
2. **Cross-Modal Attention Mechanism**: An improved cross-modal attention mechanism is proposed, using the text modality to assist in the modeling of image. Through an enhanced Transformer structure, effective cross-modal fusion is achieved, enhancing the model’s understanding and classification performance of multimodal data.
3. **Enhanced Accuracy in Event Detection**: By combining the complementary information of text, image, the model is able to detect events more accurately, especially when relying on visual cues to supplement textual information, thus improving the accuracy and robustness of event detection.

## Related work

### Uni-source methods

Uni-source methods have been widely explored in various research domains, focusing on enhancing the accuracy and efficiency of predictive models using a single source of data. These approaches leverage different techniques ranging from sentiment analysis and text classification to multimodal learning, aiming to process and analyze diverse types of information effectively. In this section, we review key works that have contributed to the development of such methods, highlighting their novel contributions and the methodologies employed.

Wang et al. [22] develop a new sentiment word mining method that utilizes three distinct wording standards and point-wise metrics, alongside a rule set model to analyze sentiment features across various linguistic components. Yilmaz et al. [23] employ a generative latent variable model and derive a generalized expectation-maximization (EM) algorithm for effective parameter learning, demonstrating computational efficiency suitable for handling large datasets. mmETM [24] captures both textual and visual information by modeling social media documents that include long text and related images, allowing it to discern between visual-representative and non-visual-representative topics. mmETM enables the model to continuously update and refine its understanding of informative textual and visual topics. Abdulkareem [25] introduces a novel Arabic Twitter corpus and explore various machine learning models, including K-Nearest Neighbour, Naïve Bayes, and Decision Trees, to develop effective tagging systems specifically tailored for the idiosyncratic nature of social media text. Hathlian ey al. [26] employ text classification methods that include comprehensive preprocessing and various machine learning techniques, focusing on tweets as our primary data source due to Twitter’s popularity and the richness of its microblogging content. LeGo-CM [27] first constructs a graph of entities extracted from the tweets, where each edge represents the co-occurrence weight between two entities, facilitating a rich relational structure. The embeddings of these graph nodes are then learned within a shared latent space, guided by approximated stationary residing probabilities computed through personalized random walk procedures. Xu et al. [28] introduce a Merged Neural Network model that employs Convolutional Neural Networks (CNNs) to extract feature representations from both text and images, effectively capturing the nuances of sentiment expressed in multimodal content. To enhance the fusion of these multimodal features, we implement a residual model and propose two strategies—Early-RMNN (Early Residual MNN) and Late-RMNN (Late Residual MNN)—which allow for the extraction of deeper and more discriminative features. Lo et al. [29] introduce a novel ‘Joint’ ranking method that capitalizes on the strengths of various ranking techniques, which, when combined with an unsupervised topic clustering model, demonstrates significant potential for uncovering topics of interest or concern within local communities, thereby assisting decision-makers in understanding public opinions while minimizing the need for extensive manual annotation. O’Halloran et al. [30] framework operates within a multilevel, contextual model, allowing for the transformation of large, geotagged textual datasets into structured, analyzable information that supports mixed methods research. The approach is specifically designed to extract critical insights from public data, such as those found in crisis informatics, enabling more effective disaster management through the analysis of extreme event reports. Tweetluenza [31] involves two key steps: first, filtering tweets to classify them into “reporting” and “non-reporting” categories based on their relevance to Influenza, and second, using a linear regression model to predict future Influenza-related hospital visits from the reporting tweets.

These uni-source approaches illustrate the diverse strategies researchers have adopted to tackle complex tasks such as sentiment analysis, topic modeling, and event prediction using a single data modality. The integration of various machine learning models, from simple classification algorithms to sophisticated deep learning architectures, demonstrates the versatility and potential of uni-source methods in handling large and varied datasets. As we move forward, these models continue to serve as the foundation for more advanced multimodal and cross-lingual approaches, further pushing the boundaries of data-driven research.

### Multi-source methods

In the era of rapidly growing data from social media and news platforms, cross-domain event detection and tracking have become essential topics in multimodal information processing. Various data sources, such as images, text, and social media activities, play a crucial role in event identification and evolution tracking. Effectively integrating information from different platforms and modalities remains a significant challenge in improving event understanding and analysis [32]. In recent years, numerous studies have introduced innovative methods and algorithms aimed at enhancing event tracking accuracy and efficiency through multimodal fusion, cross-platform collaboration, and real-time event tracking. Muhammad Waqas et al. [34,35] utilize multimodal foundation models to capture the relationships between different modalities through large-scale pre-trained deep learning models, initializing shared cross-modal representations for each modality. By combining MIL-style instance aggregation, data from different modalities can be organized into “bags,” allowing the model to learn cross-modal latent features across multiple instances, thereby enhancing few-shot generalization and improving the model’s robustness.CO-PMHT [33] integrates images and textual content from two domains, Google News and Flickr, using a semantic posterior probability to bridge the domain gap and collaboratively track events. The EventBuilder system [36] leverages Wikipedia as background knowledge to enrich event summaries with contextual information, enhancing the understanding of input events. It automatically generates real-time event summaries by visualizing a diverse range of social media activities, integrating data from platforms like Flickr and Wikipedia. Qian et al. [37] propose a novel online multimodal multiexpert learning algorithm which features a nonparametric online multimodal tracking module that learns the number of topics over time and leverages multimodal data, along with a multiexpert minimization restoration scheme that mitigates tracking drift by allowing the model to evolve backwards. Additionally, the model effectively tracks social events and captures their topic evolution, enabling a deeper understanding of multimodal topics. Petkos et al. [38] presents a novel multimodal clustering algorithm aimed at detecting social events from heterogeneous multimedia items and their associated metadata. The method leverages existing domain-specific clustering to guide the multimodal fusion and clustering process, enhancing the overall accuracy of event detection. Khalidov et al. [39] presents a novel approach to multimodal clustering using conjugate mixture models, which allow for effective handling of data gathered from multiple sensors with potentially misaligned observations. The algorithm introduces various local and global optimization techniques, as well as two initialization strategies and a consistent model selection criterion. MMSC [40] learns a shared graph Laplacian matrix that unifies different image features for clustering purposes. Additionally, a non-negative relaxation is incorporated into the method to enhance the robustness and efficiency of the image clustering process. Katragadda et al. [41] introduce three merging approaches: simple concatenation, where data from each source is combined directly; weighted aggregation, assigning different importance to each source based on reliability; and hierarchical fusion, which organizes information by assessing the relevance of events contextually.

Hare et al. [42] addresses critical challenges in this context, including the engineering and selection of features that best indicate item similarity, a feature fusion strategy that accounts for the relative importance of different features, and the construction of a sparse affinity matrix to manage large datasets. Wang et al. [43] propose a unified algorithm that integrates events identified on both platforms (I-events from Instagram and T-events from Twitter) that occur in adjacent time periods. By employing this fusion strategy, we leverage the strengths of both sources while mitigating their weaknesses, and we validate the effectiveness of our approach through empirical evaluation with real data collected from Twitter and Instagram, demonstrating a significant improvement in tracking accuracy over baseline methods. Giridhar et al. [44] utilizes an unsupervised algorithm that correlates event signals across these two social networks, addressing the challenge of corroborating information from a single network, which often yields insufficient support for detecting events.

The proposed methods and techniques provide a rich theoretical foundation and practical insights for social event tracking and analysis. Through multimodal fusion, real-time event tracking, and intelligent integration of multi-source data, researchers have gradually overcome the challenges posed by data heterogeneity across social media and news platforms. These advancements have significantly improved the accuracy and efficiency of event detection and understanding. As the scale of data continues to grow and algorithms evolve, future research in multimodal cross-domain event detection will focus on the scalability, real-time performance, and adaptability of models in real-world applications.

## Method

**Fig 1:**
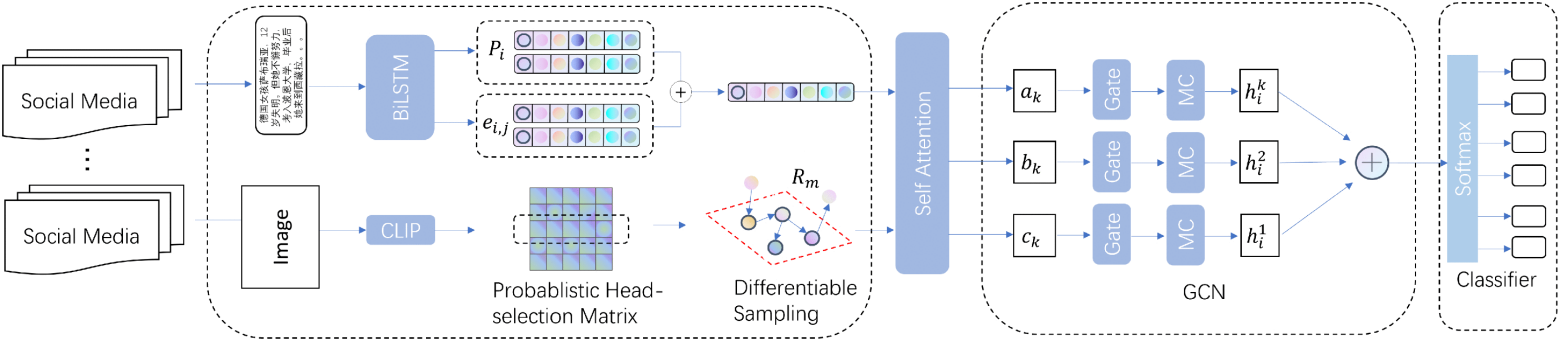
Overall architecture.

The proposed event detection model integrates dependency information using Graph Convolutional Networks (GCN) and is structured into four primary modules: the encoding layer, the graph construction module, the GCN layer, and the trigger word recognition and classification layer, as illustrated in Fig 1. Initially, word embeddings are generated using the skip-gram model, which encodes the input sentence by transforming each word into a fixed-length real-valued vector. Simultaneously, visual features are extracted from images using the pre-trained CLIP model. The graph construction module creates multi-order syntactic and dependency graphs based on the syntactic dependency tree of the sentence, with tokens as nodes and syntactic arcs as edges. The outputs from both the sentence encoding layer and the graph construction module are passed into the GCN for further modeling. After processing through the GCN, the output integrates both dependency labels and multi-hop relational information. Finally, in the trigger word recognition and classification layer, the enhanced sentence representation, enriched with dependency information from the GCN, is fed into a feed-forward neural network. This network, combined with a softmax function, classifies each word in the sentence, identifying the trigger words and categorizing them into their respective entity-based event categories (e.g., PER, LOC, ORG, OTH).

### Dataset

Our dataset consists of multimodal social media data collected from two major platforms: Weibo and Twitter-17. This dataset includes both textual and visual data, and is designed to facilitate event detection in dynamic social media environments. The Table 1 summarizes the distribution of samples across both platforms, breaking down the data into training, validation, and test sets for each event category on both Weibo and Twitter-17. The total dataset includes 6992 samples from Weibo and 5972 samples from Twitter-17, with corresponding images associated with the posts.

**Table 1:** Weibo data statistics for event detection.

Event Category	Weibo			Twitter-17		
Label	Training Set	Validation Set	Test Set	Training Set	Validation Set	Test Set
Positive	1896	632	633	1508	515	493
Neutral	1920	640	641	1638	517	573
Negative	368	149	113	416	144	168
Total	4184	1421	1387	3562	1176	1234

The Weibo dataset consists of 4184 samples in total, including both textual and visual data. Specifically, 1896 posts were collected for training, 632 posts for validation, and 633 posts for testing. The dataset also includes images, with 1920 images in the training set, 640 images in the validation set, and 641 images in the test set. In total, the Weibo data includes 4184 posts and images, providing a rich source of multimodal data for event detection.

The Twitter-17 dataset comprises 3562 samples in total. Specifically, the dataset includes 1508 tweets in the training set, 515 tweets in the validation set, and 493 tweets in the test set. Additionally, there are images associated with the tweets, with 1,638 images in the training set, 517 images in the validation set, and 573 images in the test set. Overall, the Twitter-17 dataset consists of 3562 tweets and images, making it a valuable resource for event detection in real-time social media data.

### Preprocessing methods

Before feeding the data into the event detection model, several preprocessing steps are applied to ensure that both textual and visual data are in an appropriate format for the subsequent modeling processes.

The text data undergoes several stages of preprocessing to clean and normalize the input sentences: 1. Tokenization: Sentences are first tokenized into individual words or subwords. This is done using standard tokenization techniques, where punctuation is separated from words, and compound words are split appropriately. 2. Lowercasing: All text is converted to lowercase to ensure uniformity and avoid treating the same word as different due to case sensitivity. 3. Stopword Removal: Common stopwords (e.g., “the,” “is,” “in”) are removed from the sentences, as they do not contribute significant meaning for event detection tasks. 4. Named Entity Recognition (NER): Named entities such as names of people, places, organizations, etc., are identified and tagged using an NER model. These entities are later used in the feature extraction process. 5. Dependency Parsing: A syntactic dependency tree is constructed for each sentence to capture the grammatical structure. This helps in building the graph structure where words are represented as nodes and their syntactic relationships as edges. 6. POS Tagging: Part-of-speech (POS) tagging is performed to label each word with its corresponding part of speech (e.g., noun, verb, adjective), which is then used as an additional feature in the sentence encoding process.

The visual data associated with each social media post is also preprocessed to ensure that the images are in a suitable format for the model: 1. Resizing: All images are resized to a fixed resolution to ensure uniform input dimensions. The typical size for images in our dataset is 224×224 pixels, which is compatible with most pre-trained deep learning models. 2. Normalization: Image pixel values are normalized to a range of [0, 1] or [−1, 1] based on the model’s input requirements. This normalization step ensures that the visual features are on a similar scale, making it easier for the neural network to learn effectively. 3. Data Augmentation: To increase the robustness of the model and prevent overfitting, various augmentation techniques are applied to the images during training. These techniques include random rotations, flips, and color adjustments to simulate different real-world scenarios and enhance the diversity of the dataset. 4. Feature Extraction: Pre-trained convolutional neural networks (CNNs), the CLIP model, are used to extract high-level semantic features from the images. These features are then integrated into the multimodal event detection model.

Once the text and image data have been preprocessed, the next step involves the fusion of both modalities. The textual and visual features are extracted separately, and then concatenated into a unified multimodal representation. This fusion process allows the model to leverage both the semantic content from the text and the visual context from the images for more accurate event detection.

### Sentence and image encoding module

The sentence encoding module plays a crucial role in the event detection model, converting the input target sentence $W=\{w_{1},w_{2},\ldots ,w_{n}\}$ into a sequence of real-valued vectors suitable for further processing. To accommodate a fixed input length, the sentence length *n* can be determined by truncating or padding the sentence. To capture the full semantic information of the sentence, the sentence encoding module not only relies on the word embeddings but also incorporates part-of-speech features, entity information, and contextual features, thereby overcoming the limitations of analyzing sentences in isolation.

For each word $w_{i}$, the following features are used to generate its corresponding real-valued vector $x_{i}$: 1. Word embedding vector: Each word $w_{i}$ is represented by a pre-trained word embedding vector ${\text{word}}_{i}$. Similar to previous works, this paper uses word embeddings pre-trained on the NYT corpus using the skip-gram model, obtaining the word vector through an embedding lookup. 2. Entity type embedding vector: Entities in the sentence are annotated using the BIO tagging scheme, and each entity label is mapped to a real-valued embedding vector ${\text{entity}}_{i}$, which is obtained by looking up the predefined entity embedding table. 3. Part-of-speech (POS) embedding vector: Each word also receives a part-of-speech tag (POS), and this tag is mapped to a corresponding embedding vector ${\text{pos}}_{i}$ by looking up a randomly initialized POS embedding matrix.

These features are concatenated to form the final input embedding $x_{i}$ for each word $w_{i}$, which is defined as:


$$\begin{array}{c} x_{i}=[{\text{word}}_{i};{\text{entity}}_{i};{\text{pos}}_{i}]\in {\mathbb{R}}^{d_{\text{word}}+d_{\text{entity}}+d_{\text{pos}}} \end{array}$$

where *d*_word_, *d*_entity_, and *d*_pos_ represent the dimensionalities of the word embedding, entity type embedding, and POS embedding, respectively. As a result, the input sentence *W* is transformed into a real-valued vector sequence $X=[x_{1},x_{2},\ldots ,x_{n}]$.

Next, a Bidirectional Long Short-Term Memory (Bi-LSTM) network is used to capture the contextual information for each word. Specifically, for each word $x_{i}$, the Bi-LSTM network performs both forward and backward encoding, producing the forward hidden state $p_{i}^{\to }$ and the backward hidden state $p_{i}^{\leftarrow }$, and then concatenates them to form the final contextual vector $p_{i}$:


$$\begin{array}{c} p_{i}^{\to }={\text{LSTM}}^{\to }(x_{i}) \end{array}$$

$$\begin{array}{c} p_{i}^{\leftarrow }={\text{LSTM}}^{\leftarrow }(x_{i}) \end{array}$$

$$\begin{array}{c} p_{i}=[p_{i}^{\to };p_{i}^{\leftarrow }] \end{array}$$

Here, ${\text{LSTM}}^{\to }$ and ${\text{LSTM}}^{\leftarrow }$ denote the forward and backward LSTM computations, respectively. After the Bi-LSTM encoding, the resulting contextual vector sequence $P=[p_{1},p_{2},\ldots ,p_{n}]$ is used as input to the graph convolutional network layer for the integration of dependency information and event trigger identification.

In this way, the sentence encoding module not only represents the semantics of individual words but also captures the contextual relationships between words, providing rich feature inputs for the subsequent graph convolutional network layer.

The input to the visual encoder is the image *V* associated with the post. In various visual understanding tasks, image descriptors trained using Convolutional Neural Networks (CNNs) over large datasets have shown significant effectiveness. The implicit learning of spatial layout and object semantics in the deeper layers of the network contributes to the success of these features. We employ the pre-trained CLIP (Contrastive Language-Image Pre-training) model, which is trained on a large dataset, and use the output from the visual encoding part of the model. During joint training, the parameters of the CLIP model are frozen to prevent parameter explosion. The output of the CLIP model is then passed through several fully connected layers to obtain a feature representation of the image with the same dimensionality as the text.

The visual feature representation $R_{V}$ is computed as follows:


$$\begin{array}{c} R_{V}=\phi (W_{vf}R_{CLIP}) \end{array}$$

where $R_{CLIP}$ is the feature representation obtained from the CLIP model, $W_{vf}$ is the weight matrix of the fully connected layer, and ϕ is the activation function. The textual feature representation $R_{T}$ and the visual feature representation $R_{V}$ are concatenated and passed through a fully connected layer to form a shared representation. From the shared representation, we obtain two vectors μ and σ, which can be treated as the mean and variance of the distribution of the shared representation. Furthermore, a random variable ϵ is sampled from a predefined distribution (e.g., a Gaussian distribution). The final reparameterized multimodal representation $R_{m}$ is computed as:


$$\begin{array}{c} R_{m}=\mu +\sigma \circ \epsilon \end{array}$$

We denote the encoder as $G_{enc}(M,\theta_{enc})$, where $\theta_{enc}$ represents all the parameters to be learned in the encoder, and *M* represents the set of multimedia posts. The output of the encoder for a multimedia post *m* is the multimodal representation:


$$\begin{array}{c} R_{m}=G_{enc}(m,\theta_{enc}) \end{array}$$

### Cross-modal multi-layered attention fusion

**Fig 2:**
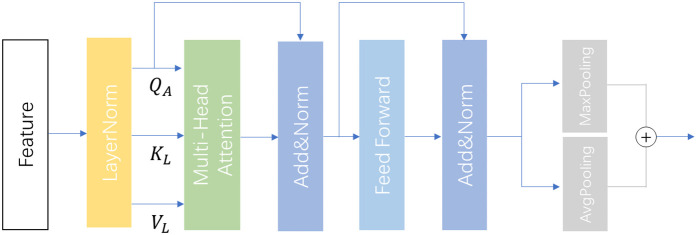
The architecture of cross-modal multi-layered attention fusion.

The self-attention mechanism plays a crucial role in enhancing the feature extraction process by capturing long-range dependencies and contextual relationships within a given modality, the architecture is illustrated in Fig 2. In this section, we leverage the Transformer model’s strengths in capturing contextual relationships to model the low-level features of each modality, thereby generating richer high-level feature representations.

To illustrate, consider the text modality. The text feature representation $X_{L}^{i}$ is first input into the Transformer, where it undergoes a multi-head self-attention process to learn the internal structure of the modality. The calculation process is as follows:

Linear Transformation of Input: The input text features are linearly transformed to produce the query ($Q_{L}$), key ($K_{L}$), and value ($V_{L}$) matrices:


$$\begin{array}{c} Q_{L}=X_{L}^{i}W_{Q},\;K_{L}=X_{L}^{i}W_{K},\;V_{L}=X_{L}^{i}W_{V} \end{array}$$

where $W_{Q}\in {\mathbb{R}}^{d_{L}^{i}\times d_{k}}$, $W_{K}\in {\mathbb{R}}^{d_{L}^{i}\times d_{k}}$, and $W_{V}\in {\mathbb{R}}^{d_{L}^{i}\times d_{v}}$ are the linear transformation weight matrices for the query, key, and value, respectively. Here, $d_{L}^{i}=d_{k}=d_{v}$ represent the dimensionalities of the respective vectors.

Attention Mechanism: The attention mechanism computes the attention scores between the query and the key, followed by applying a softmax function to normalize these scores. The weighted sum of values is then computed to produce the attention output:


$$\begin{array}{c} \text{Attention}(Q_{L},K_{L},V_{L})=\text{Softmax}\left(\frac{Q_{L}K_{L}^{T}}{\sqrt{d_{k}}}\right)V_{L} \end{array}$$

This step allows the model to focus on the most relevant parts of the input sequence, capturing contextual dependencies within the modality.

Multi-Head Attention: The multi-head attention mechanism extends this process by allowing the model to attend to multiple subspaces of the input representation in parallel. The queries, keys, and values are linearly transformed for each head, and the outputs of all heads are concatenated to form a final representation:


$$\begin{array}{c} {\text{head}}_{h}=\text{Attention}(Q_{L}W_{Q}^{h},K_{L}W_{K}^{h},V_{L}W_{V}^{h}) \end{array}$$

$$\begin{array}{c} \text{MultiHead}(Q_{L},K_{L},V_{L})=\text{Concat}({\text{head}}_{1},{\text{head}}_{2},\ldots ,{\text{head}}_{h})W_{O}^{h} \end{array}$$

where $W_{Q}^{h}\in {\mathbb{R}}^{d_{L}^{i}\times d_{k}^{h}}$, $W_{K}^{h}\in {\mathbb{R}}^{d_{L}^{i}\times d_{k}^{h}}$, $W_{V}^{h}\in {\mathbb{R}}^{d_{L}^{i}\times d_{v}^{h}}$, and $W_{O}^{h}\in {\mathbb{R}}^{hd_{v}^{h}\times d_{L}^{i}}$ are the weight matrices for each head. Here, $d_{k}^{h}=d_{v}^{h}=d_{L}^{i}/h$ represents the dimension of each head, and *h* is the number of attention heads.

Residual Connections and Layer Normalization: After the multi-head attention, residual connections are applied to the output, followed by layer normalization to stabilize and improve training:


$$\begin{array}{c} X_{L}^{\text{out}}=\text{LayerNorm}(X_{L}+\text{MultiHead}(Q_{L},K_{L},V_{L})) \end{array}$$

This step allows the model to retain the original features while integrating the contextualized information from the attention mechanism.

Feed-Forward Neural Network: The output from the attention mechanism is then passed through a feed-forward neural network composed of two linear layers with a ReLU activation function in between. The feed-forward network further processes the attended features, refining the representation:


$$\begin{array}{c} X_{L}^{\text{FFN}}=\text{ReLU}(W_{1}X_{L}^{\text{out}}+b_{1})W_{2}+b_{2} \end{array}$$

Again, residual connections and layer normalization are applied:


$$\begin{array}{c} X_{L}=\text{LayerNorm}(X_{L}^{\text{out}}+X_{L}^{\text{FFN}}) \end{array}$$

High-Level Text Feature Representation: The final output $X_{L}$ represents the high-level features for the text modality, which now encapsulate the internal relationships and contextual information learned through the self-attention mechanism and feed-forward processing.

This same procedure can be applied to the image modalities to obtain their high-level feature representations $X_{A}$ and $X_{V}$, respectively. For image, the input is processed similarly, where the image features are modeled through self-attention to capture dependencies across time. Through this process, the Transformer model not only enhances the internal representations of each modality by capturing complex, long-range dependencies but also ensures that important structural information within each modality is retained and enhanced.

### Diagram building blocks

In the Graph Convolutional Network (GCN) model, the graph construction module for image features plays a critical role in converting image data into a graph structure that can be integrated with textual information. This process consists of several stages to enable the fusion of visual and textual features, allowing for a more effective multimodal event detection system.

Given a sentence of length *n*, $W=\{w_{1},w_{2},\ldots ,w_{n}\}$, dependency parsing is performed to generate the syntactic dependency tree of the sentence. Each word in the sentence is represented as a node, and the dependency relationships between words are used to construct the dependency syntactic graph *G* = {*V*, *E*}, where $V=\{v_{1},v_{2},\ldots ,v_{n}\}$ contains the *n* nodes corresponding to each word $w_{i}$, and *E* represents the set of edges between nodes. If there exists a dependency relation between node $v_{i}$ and node $v_{j}$, a directed edge $\left(v_{i},v_{j}\right)$ is added to *E*, with a label $K(v_{i},v_{j})$ indicating the dependency type. Mathematically, this can be expressed as:


$$\begin{array}{c} G=\left\{V=\{v_{1},v_{2},\ldots ,v_{n}\},E=\{(v_{i},v_{j})|K(v_{i},v_{j})\}\right\} \end{array}$$

To facilitate the backward propagation of information, a reverse edge $\left(v_{j},v_{i}\right)$ with label $K^{\prime }(v_{i},v_{j})$ is also introduced. Additionally, self-loops $\left(v_{i},v_{i}\right)$ are added to each node to include the node’s own information. The adjacency matrix *A* of the first-order syntactic graph is then constructed, consisting of submatrices corresponding to forward, reverse, and self-loop edges:


$$\begin{array}{c} A=(\begin{array}{ccc} A_{\text{along}} & A_{\text{rev}} & A_{\text{loop}} \end{array}) \end{array}$$

where: - *A*_along_ represents the forward syntactic edges, - *A*_rev_ represents the reverse syntactic edges, and - *A*_loop_ represents the self-loops.

For higher-order syntactic graphs, the adjacency matrix is extended to capture multi-hop relationships. For a *K*-th order graph, the adjacency matrix $A_{K}$ is computed as:


$$\begin{array}{c} A_{K}=A_{\text{along}}^{K}+A_{\text{rev}}^{K}+A_{\text{loop}}^{K} \end{array}$$

where $A_{\text{along}}^{K}$, $A_{\text{rev}}^{K}$, and $A_{\text{loop}}^{K}$ represent the adjacency matrices of the *K*-th hop relationships for each type of edge. The adjacency matrices for multi-order graphs are used as the input to the subsequent graph convolutional layers.

The graph construction module also incorporates image features, which are extracted using pre-trained convolutional neural networks. These deep learning models are capable of capturing high-level semantic and structural features from images. The image input is processed through several convolutional and pooling layers, yielding a fixed-length vector ${𝐟}_{i}$ that represents the high-level semantic features of the image. These image features are then treated as nodes in the graph, alongside the textual nodes. The image features are represented as $F_{\text{image}}=\{{𝐟}_{1},{𝐟}_{2},\ldots ,{𝐟}_{m}\}$, where each ${𝐟}_{i}$ is the vector representation of an image feature.

A connection between an image feature node and a textual node is established if there is a semantic or contextual relationship between the image and the word. This relationship can be quantified using similarity measures, such as cosine similarity $\text{sim}({𝐟}_{i},{𝐰}_{j})$, which are calculated based on the image content and the textual description:


$$\begin{array}{c} \text{sim}({𝐟}_{i},{𝐰}_{j})=\frac{{𝐟}_{i}\cdot {𝐰}_{j}}{\left\|{𝐟}_{i}\|\|{𝐰}_{j}\right\|} \end{array}$$

where ${𝐟}_{i}$ and ${𝐰}_{j}$ are the feature vectors of the image and the word, respectively. If the similarity score exceeds a certain threshold, an edge is formed between the image feature node *v*_image_ and the textual word node $v_{j}$. This can be expressed as:


$$\begin{array}{c} E_{\text{image-text}}=\{(v_{\text{image}},v_{j})|\text{sim}({𝐟}_{i},{𝐰}_{j})>\theta \} \end{array}$$

where θ is the similarity threshold.

Once the graph is constructed, graph convolution operations are applied to learn node representations from the graph structure. In the GCN, each node aggregates information from its neighboring nodes to update its representation. The graph convolution operation for node $v_{i}$ can be expressed as:


$$\begin{array}{c} h_{i}^{\left(l+1\right)}=\sigma \left(\sum_{j\in 𝒩(i)}\frac{1}{\sqrt{d_{i}d_{j}}}A_{ij}h_{j}^{\left(l\right)}+W^{\left(l\right)}h_{i}^{\left(l\right)}\right) \end{array}$$

where: – $h_{i}^{\left(l\right)}$ is the feature vector of node $v_{i}$ at the *l*-th layer, – $A_{ij}$ is the adjacency matrix element between nodes *i* and *j*, – 𝒩(i) denotes the neighbors of node *i*, – $d_{i}$ and $d_{j}$ are the degrees of nodes *i* and *j*, – *W*^(*l*)^ is the weight matrix at the *l*-th layer, and – σ(·) is a non-linear activation function, such as ReLU.

Through multiple layers of graph convolution, both textual and visual information are progressively integrated into the node representations, allowing for more effective multimodal learning. This process facilitates the fusion of both image and text features, which are subsequently used for tasks such as trigger word recognition and classification in event detection. By incorporating both image and text data, the model can detect events more accurately, leveraging visual cues from images to complement the information provided by the text.

Overall, the graph construction module for image features enables the fusion of visual and textual information by building a graph structure that captures the relationships between these modalities. This multimodal representation improves the model’s ability to detect events by combining the strengths of both textual and visual data, thus enhancing the performance and robustness of the event detection model.

To better integrate the semantic features and syntactic characteristics of a sentence and to fully utilize the multi-hop information along with syntactic type label information, the module takes as input the sequence of contextual encodings $P=[p_{1},p_{2},\ldots ,p_{n}]$ from the sentence encoding layer, the multi-order syntactic graph $A_{\text{subg}}^{K}$ from the graph construction module, and the adjacency tensor *E* generated by the dependency syntactic graph. Initially, the sequence *P* produced by the sentence encoding layer is augmented with the corresponding word’s dependency relation label information. The real-valued embedding vector $e_{t_{i,j}}$ of the dependency type is mapped by the matrix $W^{T}$ to a vector of the same dimensionality as the encoding $p_{j}$, and the two vectors are concatenated to form the augmented representation of word $w_{j}$’s dependency information. This operation is mathematically expressed as:


$$\begin{array}{c} {\tilde{p}}_{j}=p_{j}+W^{T}\cdot e_{t_{i,j}} \end{array}$$

After obtaining the enhanced representation ${\tilde{p}}_{j}$, graph convolution operations are applied to the node features based on the multi-order syntactic graph. The computation is formulated as:


$$\begin{array}{c} f({\tilde{p}}_{i},A_{k})=\sigma \left(\sum_{j=1}^{n}a_{k_{i,j}}(W\cdot {\tilde{p}}_{j}+b)\right) \end{array}$$

where *W* and *b* are the weight matrix and bias term, $a_{k_{i,j}}$ refers to the element in the adjacency matrix corresponding to the forward arc in the *k* -th order syntactic graph, and σ denotes the activation function.

The *k* -th order syntactic graph $A^{K}$ consists of three n×n submatrices $a^{K}$, $b^{K}$, and $c^{K}$ that represent the forward, reverse, and self-loop syntactic relations, respectively. To effectively leverage the syntactic features, the formula (7) is applied to each of the three submatrices to obtain the corresponding feature representations for each order of the syntactic graph. The feature representation $h_{i}^{K}$ for each candidate trigger word $w_{i}$ is computed as:


$$\begin{array}{c} h_{i}^{K}=f({\tilde{p}}_{i},a^{K})⊕f({\tilde{p}}_{i},b^{K})⊕f({\tilde{p}}_{i},c^{K}) \end{array}$$

where ⊕ represents element-wise addition. This equation computes a consolidated feature representation $h_{i}^{K}$ for each word $w_{i}$ by aggregating the features from the three submatrices of the *k* -th order syntactic graph.

Finally, to integrate the multi-hop information, the multi-order feature representations $h_{i}^{K}$ for each word $w_{i}$ are aggregated to obtain the final fused feature representation. The aggregation process is formulated as:


$$\begin{array}{c} h_{i}=\sum_{K=1}^{K_{\text{max}}}h_{i}^{K}\;(9) \end{array}$$

where *K*_max_ denotes the maximum order of the syntactic graph. The final representation $h_{i}$ of each word incorporates the fused multi-hop information, which enables more accurate event detection and trigger word recognition.

### Classification module

After obtaining the fused representation of features from the graph convolution module that integrates dependency information across all modalities, these fused features are passed through a fully connected layer for the final trigger word recognition and classification. This process can be modeled as follows:


$$\begin{array}{c} p(t|h)=\text{softmax}(W_{t}\cdot h+b_{t}) \end{array}$$

where $W_{t}$ is the weight matrix that transforms the fused feature representation *h* into scores for each event label, and $b_{t}$ is the bias term. The softmax function, commonly used in multi-class classification tasks, transforms the scores into a probability distribution, representing the likelihood of each event type. The predicted entity label (trigger type) is the one with the highest conditional probability, which is selected as the corresponding candidate trigger word.

In this study, to align with the characteristics of multimodal social media data, the event trigger recognition and classification task is implemented as a Multimodal Named Entity Recognition (MNER) task. Identifying specific entities—such as Person (PER), Location (LOC), Organization (ORG), and Others (OTH)—serves as the primary mechanism for anchoring events in dynamic streams like Twitter and Weibo. Therefore, the “event types” produced by the classification layer correspond to these entity categories, following the standard BIO tagging scheme. This mapping allows the model to treat these key entities as the structural triggers of an event, ensuring consistency between the architectural design and the evaluation metrics presented in the experimental section.

In this way, the model classifies the input data by selecting the event type with the highest probability, and this event type is considered the predicted trigger word. By doing so, the model effectively identifies the trigger words for event detection from the fused multimodal features.

### Evaluation indicators

In the construction of the dataset, all posts were simulated using a client-server architecture. Specifically, each post from Twitter and Weibo is pushed from the server to the client based on the post’s creation time. Posts from Twitter are collected in micro-batches at one-minute intervals, while posts from Weibo are collected by periodically executing queries that return results from the same time period. Within the first five minutes, all posts are compared with those posted within the last hour to assist with real-time event detection.

To evaluate the performance of various event detection strategies, we present the analysis of the detected events on both Twitter and Weibo in Table 1. The table shows the results for different merging strategies: merge during graph generation, merge during graph filtering, and merge post-clustering. The number of detected events in the table represents the total number of events identified by each method in each scenario.

The performance of the model is assessed using precision, recall, and F-score, which are calculated using the following formulas:

- Precision is computed using the formula in Equation (4). It represents the proportion of detected events that are actual true events. Specifically, precision is calculated as the ratio of detected events to the sum of detected events and noise (false positives).
  $$\begin{array}{c} \text{Precision}=\frac{\text{Number of Detected Events}}{\text{Number of Detected Events}+\text{Number of Noise Events}} \end{array}$$
- Recall is calculated using Equation (5) and reflects the proportion of actual events that were successfully detected by the model. Recall is the ratio of detected events to the total number of true events.
  $$\begin{array}{c} \text{Recall}=\frac{\text{Number of Detected Events}}{\text{Total Number of True Events}} \end{array}$$
- F-score is calculated using Equation (6) and is the harmonic mean of precision and recall. The F-score balances the trade-off between precision and recall, providing a single metric that incorporates both.
  $$\begin{array}{c} F-\text{score}=\frac{2\times \text{Precision}\times \text{Recall}}{\text{Precision}+\text{Recall}} \end{array}$$

The total number of true events is computed as the sum of the detected events and the undetected events. Undetected events refer to actual events that occurred during the observation period but were not identified by any method. By comparing the detected events to the true events, the model’s ability to identify relevant events while minimizing false positives and false negatives can be evaluated.

In the analysis, special attention is given to the impact of various merging strategies (e.g., merge during graph generation, merge during graph filtering, and merge post-clustering) on precision, recall, and F-score. These merging strategies are designed to enhance the accuracy and comprehensiveness of event detection by reducing noise and improving the detection of relevant events.

Furthermore, by observing the variations in precision, recall, and F-score across different strategies, insights can be gained into the effectiveness of each merging approach in different data streams (Twitter and Weibo). This evaluation helps to identify the strengths and weaknesses of each method, providing a foundation for optimizing event detection algorithms in social media data, which is often noisy and dynamic. Such an analysis allows for continuous improvement of detection systems, leading to better performance in real-world applications.

## Experiment

### Implementation details

The experiment was deployed on a server running the Ubuntu 18.04 operating system, equipped with an Intel Xeon Gold 5118 CPU and an NVIDIA Tesla V100 GPU, providing robust computational power for the experiment. The software environment utilized Python version 3.8.13, PyTorch version 1.12.1, and CUDA 11.4 to support GPU acceleration. To improve data quality and meet the requirements of subsequent tasks, several data preprocessing steps were applied to the collected raw data. Duplicate and non-textual data were removed, and irrelevant information was manually identified and deleted to ensure the precision of the collected text. Additionally, punctuation marks and other special characters that have no impact on the semantics were removed. To further enhance the precision of the data, stop words in the Weibo text data were filtered out with reference to the Harbin Institute of Technology stop word list, reducing the impact of redundant vocabulary on sentiment analysis.

In terms of model configuration, we carefully selected a set of hyperparameters to ensure optimal performance. The word embeddings used for sentence encoding were set with a dimension of 300, while entity type embeddings and part-of-speech embeddings were set at 100 dimensions. The Bi-LSTM layer, used to capture contextual information, had 256 hidden units. For the Graph Convolutional Network (GCN) layers, we set the hidden units to 512 with 3 layers, enabling the model to capture complex syntactic relationships. The dropout rate was set to 0.3 to mitigate overfitting, and the learning rate for the optimizer was set to $1e^{-4}$, which was found to work well during training. The batch size for training was set to 32, and the model was trained for 20 epochs. Additionally, the number of attention heads in the cross-modal attention mechanism was set to 8, which allows the model to attend to multiple subspaces in parallel. To facilitate the fusion of multimodal data, a similarity threshold θ of 0.7 was chosen for image-text connections, ensuring a strong relationship between the features. These hyperparameters were chosen based on preliminary experiments and tuned to balance performance and computational efficiency.

### Validation

One critical issue that must be addressed is the determination of whether a detected “event” is real. To accomplish this, we generate ground truth for the detected events by leveraging news stories that were published during the period in which the event is active. Specifically, once an event is detected, all the words and phrases within the event cluster are used to query news aggregators such as Google News, Bing News, and Yahoo News for a minimum of two hours after the event’s detection. While the news articles collected during this process may not be exhaustive, they are sufficient to identify news stories that are relevant to the event. These detected events are then annotated by three independent annotators who use the news stories as a reference to assess the relevance and authenticity of the event. The final label for each event cluster is determined by a majority vote, with each event being classified as either a true event or not.

The inter-annotator agreement quantifies the level of consensus between multiple annotators. In our case, the annotator agreement is 0.67, which is considered substantial according to established guidelines. It is important to note that it is not feasible to identify all events mentioned on social media, nor is it possible to detect every event occurring in the world at a given time. Thus, our study focuses on evaluating the efficiency of different models based on the total number of true events that actually occurred and the amount of noise present, where noise refers to events that are incorrectly identified as real events but are not true occurrences in the real world.

To further verify the effectiveness of our proposed model in event detection tasks, we conducted comparative experiments with existing methods, as shown in the Table 2. The table lists the performance of various text and text plus image methods on two datasets. It covers multiple event types including person, location, organization, and other, as well as overall performance metrics such as Precision, Recall, and F1 score. The first group of methods includes BiLSTM-CRF, BERT-CRF, and span-based named entity recognition models (such as BERT-SPAN and RoBERTa-SPAN), all of which utilize only raw text. The second group consists of recent multimodal approaches that incorporate both text and its corresponding images: UMT, UMGF, MNER-QG, R-GCN, and CAT-MNER. Experimental results demonstrate that multimodal methods generally outperform traditional text-only approaches, particularly on the Twitter-2017 dataset. For instance, in the “PER” category, BERT-CRF achieves an F1 score of 90.66% on Twitter-2017, while CAT-MNER achieves an F1 score of 90.47%. On the Weibo dataset, CAT-MNER also performs well, attaining an F1 score of 84.70%, especially in the “LOC” category. Although performance varies on the Weibo dataset, several multimodal methods still outperform traditional text-based methods across multiple categories. The experiments also show that models incorporating multimodal auxiliary knowledge maintain high precision even when the annotation quality of the dataset is lower, particularly on the Twitter-2017 dataset, which benefits from more complete and standardized annotation methods. Overall, the experimental results validate the superiority of multimodal approaches in MNER tasks, showcasing their robustness and efficiency in handling complex tasks.

**Table 2:** Performance comparison on the Weibo and Twitter-17 datasets.

Methods	Weibo							Twitter-2017						
	Single Type(F1)				Overall			Single Type(F1)				Overall		
	PER	LOC	ORG	OTH.	Pre.	Rec.	F1	PER	LOC	ORG	OTH.	Pre.	Rec.	F1
Text														
BiLSTM-CRF^†^	76.77	72.56	41.33	26.80	68.14	61.09	64.42	85.12	72.68	72.50	52.56	79.42	73.43	76.31
BERT-CRF^‡^	85.37	81.82	63.26	44.13	75.56	73.88	74.71	90.66	84.89	83.71	66.86	86.10	83.85	84.96
BERT-SPAN^‡^	85.35	81.88	62.06	43.23	75.52	73.83	74.76	90.84	85.55	81.99	69.77	85.68	84.60	85.14
RoBERTa-SPAN^‡^	87.20	83.58	66.33	50.66	77.48	77.43	77.45	94.27	86.23	87.22	74.94	88.71	89.44	89.06
Text+Image														
UMT	85.24	81.58	63.03	39.45	71.67	75.23	73.41	91.56	84.73	82.24	70.10	85.28	85.34	85.31
UMGF	84.26	83.17	62.45	42.42	74.49	75.21	74.85	91.92	85.22	83.13	69.83	86.54	84.50	85.51
MNER-QG	85.68	81.42	63.62	41.53	77.76	72.31	74.94	93.17	86.02	84.64	71.83	88.57	85.96	87.25
R-GCN	86.36	82.08	60.78	41.56	73.95	76.18	75.00	92.86	86.10	84.05	72.38	86.72	87.35	87.11
CAT-MNER	88.04	**84.70**	68.04	52.33	78.75	78.69	78.72	94.61	88.40	88.14	**80.50**	90.27	90.67	90.47
Ours	**88.34**	84.22	**70.15**	**52.34**	**79.21**	**79.45**	**79.33** ^*^	**96.46**	**89.89**	**89.03**	79.62	**90.86**	**92.01**	**91.43** ^*^
	±0.02	±0.12	±0.36	±0.98	±0.63	±0.22	±0.06	±0.02	±0.68	±0.53	±2.25	±0.16	±0.07	±0.09

Despite the strong performance of the proposed method across most event types, particularly in the “PER” and “ORG” categories on the Weibo and Twitter-17 datasets, where it achieves F1 scores of 96.46% and 89.03%, respectively, there are still limitations in its performance for certain categories. For example, in the “LOC” category of the Weibo dataset, although the model achieves an F1 score of 84.22%, it falls short of the “CAT-MNER” method (F1 = 84.70%), indicating that the detection of location-based events remains challenging. This could be due to the complex contextual and background information associated with location-related events, which makes the semantic alignment between image and text features difficult, thus affecting the final detection results. Additionally, the F1 score for the “OTH.” category is 52.34%, reflecting the increased difficulty of event detection in this category. This category typically involves non-standardized or ambiguous event types, making the model more prone to misclassification, particularly when there is insufficient semantic alignment between images and text. Misclassifications are primarily seen in the confusion between location and other event categories, especially when geographic names are similar to other entities, such as organizations or persons, leading to misclassification into the “OTH.” category. While the integration of multi-level syntactic dependency information through the Graph Convolutional Network (GCN) effectively captures syntactic relationships, the propagation of information through complex multi-hop dependencies may be limited, causing some critical event features to be inadequately extracted. Overall, while the proposed method achieves excellent results for most event types, further optimization is needed for handling complex event categories, particularly those involving location and the integration of multimodal information.

This methodology provides a robust framework for evaluating event detection systems, particularly in dynamic and noisy data streams such as social media. By comparing different models using these metrics, we gain insights into the strengths and limitations of each approach, facilitating the improvement of event detection techniques for future applications.

### Result and analysis

In this section, we evaluate the performance of our proposed framework by analyzing its core hierarchical stages: Event Detection at Onset (EDO), Stream Merging, Graph Filtering, and Graph Clustering. Unlike traditional single-source methods, our system treats EDO as the initial detection backbone. The following analysis demonstrates how the integration of Twitter and Weibo streams evolves through these stages to achieve the final detection efficacy presented in Table 5.

When applying the Event Detection at Onset model to both Twitter and Weibo datasets independently, a total of 291 events were identified from Twitter, while 85 events were detected from Weibo. The execution time for processing a 1-minute micro-batch of Twitter data was 42 seconds, whereas the same micro-batch for Weibo data took only 23 seconds. Although Weibo exhibited faster processing times, the total number of detected events was significantly lower compared to Twitter, with Weibo also generating a larger number of noise clusters, i.e., clusters that do not correspond to actual events. This suggests that when Weibo is used as the sole information source for breaking news, it may not be a reliable platform due to its low volume of valid events, coupled with an overwhelming amount of noise.

During the graph generation phase, merging the data streams from both Twitter and Weibo did not result in the identification of any additional events compared to using Twitter data alone, while the execution time increased by only one second. Our analysis indicates that the data derived from Weibo is relatively insignificant when compared to the data generated from Twitter, and thus, it is largely considered noise by the Event Detection Onset model. However, in subsequent models, such as graph filtering and graph clustering, combining both data streams was able to identify events that were missed by either Twitter or Weibo when used independently. This demonstrates the potential benefit of merging multiple data streams to enhance event detection.

In the graph filtering phase, a total of 332 events out of 347 verifiable events were detected within 44 seconds by combining the Twitter and Weibo datasets. The combined approach resulted in the identification of 39 additional events over the use of Twitter alone. However, it also generated 52 unverified events, which can be classified as noise. The number of noisy events was greater than those identified when using either Twitter or Weibo independently. Despite the increase in noise, we believe that the detection of additional events through the combination of data streams compensates for this minor increase in noise. Although precision decreased when combining multiple sources, recall saw a significant improvement compared to using Twitter or Weibo alone. Moreover, the F-Score also increased, reflecting the model’s improved overall performance when utilizing two data streams.

In the graph clustering phase, 342 out of 347 verifiable events were successfully identified. This model demonstrated the highest precision, identifying over 98% of the verifiable events in the dataset. However, it also resulted in a larger number of noise events, as events from Twitter and Weibo that were misclassified as actual events during the previous phase were passed on for clustering without further filtering. This led to the generation of additional noisy clusters. Nonetheless, graph clustering was still the most effective model for event detection in terms of identifying a higher number of valid events, despite the increased generation of noise clusters. The model required two additional seconds to execute compared to the merge during graph filtering phase, which is a trade-off for improved event detection performance.

Graph clustering was more effective in identifying a larger number of events than using a single stream or merging the streams at the initial stage of the detection process. The primary trade-off with this approach is the additional execution time, which is a necessary cost for achieving higher event detection performance. The increased execution time, while contributing to higher precision, needs to be considered when evaluating the suitability of this model for real-time applications, where processing speed may be critical. The superior performance of the graph clustering phase, which effectively balances recall and precision, represents the final output of our proposed method, as reflected in the results presented in Table 5.

Table 3 presents the clusters of events detected by our system, along with the actual events identified from the Twitter and Weibo platforms. Each event cluster generated by the system was manually evaluated, with annotators labeling the clusters as either true events or not based on the words that formed the cluster and the corresponding tweets or Weibo posts. The evaluation process confirmed that all the events detected by the system were recognized as novel events. However, certain words required to fully identify the context of some clusters were missing due to their low scores during the graph pruning process. This caused some event clusters to lack coherence. To improve understanding, both the tweets and context associated with the event clusters were provided, offering a clearer interpretation of the detected event clusters.

**Table 3:** Events detected using Twitter and Weibo.

Event Clusters from EDO	Event
*Twitter*	
emergency, court, security, building	Courthouse in Dearborn cleared due to security concern
bounty, concealment, officer, search, agency	Agency offers reward for information on suspect hiding in Cuba
cleared, police, school, area, danger, explosive	Educational institution in the Bronx cleared over explosive risk
travel, incident, shooting, airport, houston	Incident at Houston’s airport involving gunfire
incident, research, substance, lab, swiss	Lab incident in Switzerland with hazardous material
weather, alert, storm, alert, region, severe	Severe weather alert for Illinois due to storms
storm, city, alert, severe, weather, memphis	Severe weather conditions in Memphis, tornado alert issued
*Weibo*	
protest, support, community, demonstration	Community support for a protest distribution hub
policy, detention, strike, release, facility	Call for the release of detainees in a facility
incident, location, fire, emergency	Emergency situation with fire in a specific location

To verify the real-world utility of our hierarchical merging pipeline, we compare its detection latency against traditional news media in Table 4. The detected events in the dataset are categorized into six distinct categories based on their descriptions in Wikipedia. Events that do not have a corresponding Wikipedia entry are manually classified by three annotators. This approach ensures that even less-known or emerging events, which might not yet have reached widespread attention, are appropriately classified. Table 4 presents a breakdown of the total number of events detected in the dataset, alongside the average time required by different models and traditional news media to detect various types of events.

**Table 4:** Types of events, detection and verification time.

Type of Event	Events in Dataset	Detection Time (mins)	Time difference between Social Media and News (mins)
Armed Conflicts & Attacks	131	7.04	19.25
Arts, Culture & Entertainment	16	13.49	−33.38
Business & Economy	12	9.34	−6.47
Disasters & Accidents	97	7.19	12.88
Law, politics & Scandals	18	9.45	−3.07
Science & Technology	8	9.28	−8.49
Sports	65	6.79	24.77

The Event Detection Onset model proves highly effective in identifying emerging events globally, particularly in the categories of armed conflicts and attacks, as well as disasters and accidents. The model detects these events within 7.04 minutes and 7.19 minutes, respectively, which is considerably faster than the time taken by traditional news media. Traditional media outlets require approximately 19.25 minutes to report news regarding armed conflicts and attacks, and 12.88 minutes for accidents and disasters. The faster detection by the model is primarily due to the nature of these events, which often break first on social media platforms before being picked up by traditional news outlets. This pattern reflects the speed at which social media platforms disseminate information compared to the slower, more deliberate reporting processes typical of traditional media. Sports events represent another category in which social media leads traditional news outlets in terms of reporting speed. This is largely due to the instantaneous nature of information generation on social media, where updates about sports events are posted in real-time by users. The lag in traditional media can be attributed not to the ability to gather information, but to the publication processes, which tend to prioritize comprehensive reports rather than minute-by-minute updates. As a result, sports events are often covered more quickly and extensively on platforms like Twitter, where users are constantly updating the public on the progress of ongoing events.

Among the different types of events, Twitter generally leads in terms of being the primary platform for discussions, particularly for breaking news, political events, and disaster-related incidents. However, Weibo contributes more significantly in the realm of multimedia content, such as images. Weibo’s strength lies in its ability to serve as a platform for rich media content that complements textual information, making it particularly valuable in situations where visual context is crucial for understanding the event.

Table 5 shows the detection effectiveness results for each method, including Precision (P), Recall (R), and F1 score. As shown in the table, our method outperforms the others in all three metrics, particularly in Recall (R), which reaches 0.8333, significantly higher than the other methods. The KEM method also performs well in Recall, but its Precision (P) and F1 score are relatively lower. Other methods, such as HashtagPeaks and RTED, show higher Precision, but their Recall and F1 scores fall short compared to our model. Overall, our method achieves the best detection effectiveness, with a clear advantage in improving Recall and F1 scores.

**Table 5:** Modified detection effectiveness results.

Method	P	R	F1
HashtagPeaks	0.5310	0.3867	0.3461
RTED	0.6261	0.4033	0.3880
eTrack	0.6184	0.4100	0.3908
EECM	0.6333	0.4233	0.4053
KEM	0.6633	0.7267	0.5934
Ours	0.7391	0.8333	0.6832

## Conclusion and discussion

In this study, we propose a novel framework for real-time event detection by integrating multiple data streams through time-evolving graphs. The model is designed to handle diverse data sources, such as social media platforms, by dynamically merging information from Twitter, Weibo, and other potential sources. We present three distinct approaches to merge data streams: Graph Generation, Graph Filtering, and Post-Cluster Merging. Each approach is tested and evaluated based on its performance in terms of event detection accuracy, computational efficiency, and real-time applicability. Experimental results indicate that combining information from multiple streams not only improves the precision and recall of detected events but also provides a more comprehensive understanding of dynamic events unfolding across different platforms. Moreover, the proposed framework can be extended to incorporate additional data sources, such as Instagram, Snapchat, and RSS feeds, to further enhance event detection capabilities. The incorporation of multimodal data, including images, holds potential to improve the quality of detected events by providing richer context. Additionally, future extensions of the model may involve automated event validation using newswire data to enhance the reliability of the detection process.

In real-world applications, this model has significant potential in various fields such as crisis management and misinformation detection. In crisis management, real-time event detection can provide early warnings of natural disasters, terrorist attacks, or large-scale public health emergencies by aggregating information from multiple social media platforms and news sources. This allows emergency response teams to act quickly and make informed decisions. In the context of misinformation detection, the model can identify the spread of fake news or malicious content across platforms, helping to detect and mitigate the impact of false information in real-time. By leveraging multimodal data, such as images, the model can also detect visual cues of misinformation, thereby enhancing its ability to identify misleading content. Overall, the proposed framework has broad applications in monitoring, analyzing, and responding to real-time events in various domains.
